# Differences between ketamine’s short-term and long-term effects on brain circuitry in depression

**DOI:** 10.1038/s41398-019-0506-6

**Published:** 2019-06-28

**Authors:** Natalia Gass, Robert Becker, Jonathan Reinwald, Alejandro Cosa-Linan, Markus Sack, Wolfgang Weber-Fahr, Barbara Vollmayr, Alexander Sartorius

**Affiliations:** 1 Research Group Translational Imaging, Department of Neuroimaging, Central Institute of Mental Health, Medical Faculty Mannheim, Heidelberg University, Mannheim, Germany; 2 Department of Psychiatry and Psychotherapy, Central Institute of Mental Health, Medical Faculty Mannheim, Heidelberg University, Mannheim, Germany; 3 Research Group In Silico Pharmacology, Central Institute of Mental Health, Medical Faculty Mannheim, Heidelberg University, Mannheim, Germany; 4 Research Group Animal Models in Psychiatry, Department of Psychiatry and Psychotherapy, Central Institute of Mental Health, Medical Faculty Mannheim, Heidelberg University, Mannheim, Germany

**Keywords:** Neuroscience, Depression

## Abstract

Ketamine acts as a rapid clinical antidepressant at 25 min after injection with effects sustained for 7 days. As dissociative effects emerging acutely after injection are not entirely discernible from therapeutic action, we aimed to dissect the differences between short-term and long-term response to ketamine to elucidate potential imaging biomarkers of ketamine’s antidepressant effect. We used a genetical model of depression, in which we bred depressed negative cognitive state (NC) and non-depressed positive cognitive state (PC) rat strains. Four parallel rat groups underwent stress-escape testing and a week later received either S-ketamine (12 NC, 13 PC) or saline (12 NC, 12 PC). We acquired resting-state functional magnetic resonance imaging time series before injection and at 30 min and 48 h after injection. Graph analysis was used to calculate brain network properties. We identified ketamine’s distinct action over time in a qualitative manner. The rapid response entailed robust and strain-independent topological modifications in cognitive, sensory, emotion, and reward-related circuitry, including regions that exhibited correlation of connectivity metrics with depressive behavior, and which could explain ketamine’s dissociative and antidepressant properties. At 48 h ketamine had mainly strain-specific action normalizing habenula, midline thalamus, and hippocampal connectivity measures in depressed rats. As these nodes mediate cognitive flexibility impaired in depression, action within this circuitry presumably reflects ketamine’s procognitive effects induced only in depressed patients. This finding is especially valid, as our model represents cognitive aspects of depression. These empirically defined circuits explain ketamine’s distinct action over time and might serve as translational imaging correlates of antidepressant response in preclinical testing.

## Introduction

Major depressive disorder (MDD) is a globally prevalent psychiatric illness with cognitive, affective, and somatic symptoms, including negative emotional states, cognitive impairments, anxiety, anergia, and anhedonia^1^. These symptoms correlate with the aberrant connectivity in brain circuits, suggesting it to represent a mechanistic background of depression^2,3^, and implying that MDD is a brain circuit disorder^3^. Recent evidence indicates that ketamine, an NMDA receptor antagonist with rapid antidepressant effects sustained for 7 days^4^, has high potential to normalize disrupted network connectivity, proposing it as the mechanism of action^5^. However, ketamine induces a complex response unfolding distinct influences over time, producing antidepressant effects as fast as 30 min after administration at 0.5 mg/kg in humans^6^ and at a comparable dosage of 10 mg/kg in rat models^7,8^. Yet at the same time, ketamine induces psychotomimetic and dissociative side effects for the first 2 h^9^. Interestingly, a recent study demonstrated that ketamine’s dissociative effects are not entirely discernible from therapeutic action and might even predict a more robust and sustained antidepressant efficacy^10^. This dual action might result from affecting the same multifunctional regions, which are involved in both effects. Facing ketamine’s complex action over time, it is important to disentangle the mechanistic network alterations underlying ketamine’s activity at different time points.

So far, none of the neuroimaging studies on ketamine directly compared its effects across two time points. Moreover, they were conducted on healthy subjects^11–13^, although ketamine’s effects in depressed brain may differ. Those few recent studies that investigated ketamine effects in depressed patients used seed or global connectivity analyses^5,14^. However, since any brain function depends on complex network organization, a quantitative analysis of the brain network architecture using more sophisticated graph theory is highly advantageous, as it dissects network topology and identifies aberrant reconfigurations in functional brain networks.

To address these unresolved issues, we used graph analysis to dissect the relevant circuits for short-term and long-term effects of ketamine in a well-established genetical rat model of depression built upon learned helplessness paradigm in which rats are bred based on their susceptibility to develop stress-escape behavior in operant boxes^15^. The rat strain failing to escape when exposed to electrical footshocks (negative cognitive (NC) state strain) reflects the cognitive dysfunction characteristic for depressive behavior^16^, models treatment-resistant depression^15^ and posttraumatic stress disorder (PTSD)^17^. The term “NC state strain” reflects the pessimistic response bias in cognitive tasks observed in this strain^16,18^. The positive cognitive (PC) state strain displaying escape behavior represents non-depressed phenotype^15^. The model has a high face, construct, and predictive validity^15^. One of its major advantages is that it represents cognitive aspects of depression, in which events are considered negative and uncontrollable, resulting in feelings of anxiety and helplessness. Animal models have the crucial advantage of providing homogenous groups in contrast to human studies, which usually recruit medicated patients with complex and greatly heterogeneous symptoms often influenced by comorbidities with other psychiatric disorders. In addition, animal models are used in preclinical studies to support drug assessment and approvals.

First, we investigated behavioral deficits and network topological alterations in depressed rats to characterize animal model before ketamine administration. Then, we acquired functional magnetic resonance imaging (fMRI) scans at 30 min and 48 h post-ketamine and calculated network properties to distinguish ketamine’s short-term and long-term impact on brain circuitry.

## Methods and materials

### Experimental design

**Fig. 1:**
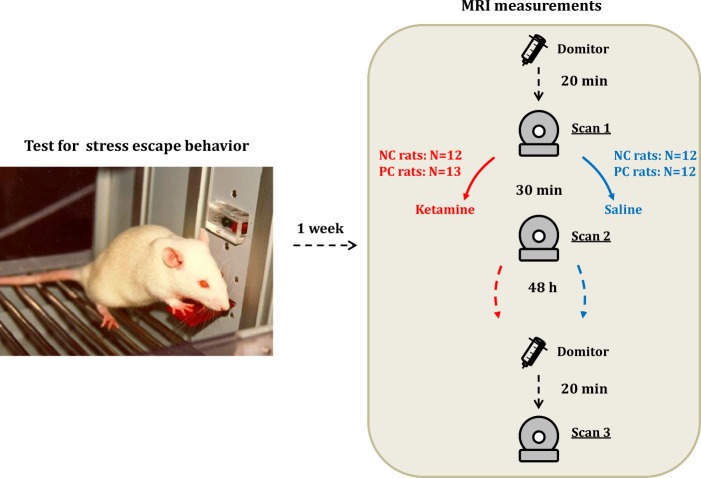
A scheme of the experimental timeline. The fMRI sessions followed test for escape behavior one week later. They included the baseline preinjection fMRI measurement (scan 1) acquired 20 min after the start of domitor continuous infusion, i.e., after stabilization of respiratory and cardiac rhythms, and the second fMRI scan at 30 min a"fter ketamine/saline injection (scan 2). After 48 h we repeated the measurement (scan 3). NC - negative cognitive state strain, PC - positive cognitive state strain. The image of the rat in operant box is reproduced from “Neurobiologie depressiver Störungen” by Psychiatrie und Psychotherapie Up2date 2010, with permission from Georg Thieme Verlag.

Four parallel groups of NC and PC male rats (*n* = 49; 82–83rd genetical generations; Sprague-Dawley; 8 weeks old; 282–414 g) underwent an escape test and 1 week later received a subcutaneous injection of either S-ketamine at 10 mg/kg (Ketanest, Pfizer Pharma GmBH, Berlin, Germany) (12 NC, 13 PC rats) or saline (12 NC, 12 PC rats), both at volume 2 ml/kg (Fig. 1). We selected 10 mg/kg dose based on previously reported antidepressant-like effects in rats^19,20^. This dose also results in a similar range of peak concentrations as 0.5 mg/kg dose in human studies^21^. We used S-ketamine, since so far it is the only clinically tested enantiomer that was shown to exert rapid antidepressant effects in patients^22^. R-ketamine effects were tested only in rodents^23^, and it remains to be demonstrated whether it has similar antidepressant properties, as S-ketamine in clinical population. Considering the similarity in the pharmacokinetic-pharmacodynamic properties of ketamine in rats and humans^24,25^, and in the timing of its antidepressant action^7,8^, we selected 30 min and 48 h for MRI measurements.

Due to the exploratory nature of this study, no formal power or sample size estimation was performed, but the group sizes (*n* = 12–13 per group) are toward the high end of the range typically used in animal fMRI experiments.

The rats were housed in plastic cages, two rats per cage, at constant temperature of 22 °C and 12-h light-dark cycle (lights on at 07:00 am). Food and water were available ad libitum. The rats were killed at the end of the experiments. We conducted experiments according to the regulations covering animal experimentation within the European Union (European Communities Council Directive 86/609/EEC) and within the German Animal Welfare Act, and were approved by the German animal welfare authorities (Regierungspräsidium Karlsruhe).

### Test for escape behavior

Behavioral experiments were performed, as before^26^, and consisted of an escape paradigm, in which animals could avoid electrical footshocks by pressing lever in operant conditioning chambers (for details see Supplement).

**Fig. 2:**
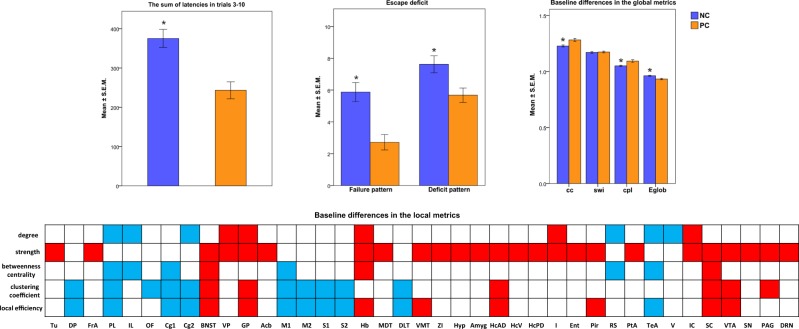
*Top left*: Significant differences in the sum of latencies to terminate footshock in the behavioral test between the NC and PC rats. *Top middle*: Significant differences in the failure and deficit pattern behavior between the NC and PC rats. *Top right*: Baseline preinjection differences in the graph analytical global properties between the NC and PC strains. Abbreviations: cc global clustering coefficient, cpl characteristic path length, Eglob global efficiency, swi small-world index. *Low*: Baseline (scan 1) differences in the graph analytical local properties between NC and PC rats. Blue color depicts values lower in the NC group, as compared to the PC group, red color—higher values in the NC group, as compared to the PC group. Asterisk indicates statistically significant difference (*p* < 0.05). Brain regions: Acb nucleus accumbens; Au auditory cortex; BNST bed nucleus of stria terminalis; CPu caudate-putamen; Cg1 cingulate cortex, area 1; Cg2 cingulate cortex, area 2; DLT dorsolateral thalamus; DP dorsal peduncular cortex; DRN dorsal raphe nuclei; Ent entorhinal cortex; FrA frontal association cortex; Hb habenula; HcAD hippocampus, anterodorsal; HcPD hippocampus, posterodorsal; HcSDG hippocampus, subiculum and dentate gyrus; Hyp hypothalamus; I insular cortex; IC inferior colliculus; IL infralimbic cortex; M1 primary motor cortex; M2 secondary motor cortex; MDT midline dorsal thalamus; OF orbitofrontal cortex; PAG periaqueductal gray; Pir piriform cortex; PL prelimbic cortex; PtA parietal association cortex; RS retrosplenial cortex; S1 primary somatosensory cortex; S2 secondary somatosensory cortex; SC superior colliculus; Sept septum; TeA temporal association cortex; V visual cortex; VMT ventromedial thalamus; VP ventral pallidum; VTA ventral tegmental area; ZI zona incerta

Using nonparametric Kruskal–Wallis test due to non-normal distribution of the data, we calculated difference between NC and PC groups for the following parameters: sum of latencies for trials 3–10, number of failures to terminate shock (*failure pattern*), and number of trials not stopped within the first 20 s (*deficit pattern*)^27^ (significance level at *p* < 0.05). We confirmed previously observed escape deficits in NC rats (Fig. 2, see details in Supplement).

### MRI acquisition and preprocessing

The resting-state fMRI (rs-fMRI) experiments were carried out at 9.4 T MRI scanner (Bruker BioSpec, Ettlingen, Germany) under anesthesia, as previously done^26^. The current data represent a newly acquired MRI dataset, not described in any of our previous studies^26,28,29^. The anesthetic regime was identical to the one described previously^26^. The rats were initially anesthetized with 4% isoflurane (Baxter Deutschland GmbH, Unterschleissheim, Germany) in a mixture of N_2_ (70%) and O_2_ (30%); after positioning in the scanner isoflurane level was reduced to 2.5% and medetomidine (Domitor, Janssen-Cilag, Neuss) was first injected as a bolus (0.5 ml, 0.07 mg/kg, s.c.) and then after a 10 min period of a slow discontinuation of isoflurane administration (reduction by 0.5% every 2 min before switching off)—as a continuous infusion (0.28 mg/kg/h). The sedation depth was monitored via recording the physiological (respiratory and cardiac) parameters throughout the experiment. Breathing and cardiac signals were recorded (10-ms resolution) using the signal breakout module (Small Animal Instruments Inc., NY, USA) and the four-channel recorder (Velleman^®^ N.V., Gavere, Belgium). The physiological parameters stabilized at 15 min after the start of a continuous medetomidine infusion and stayed stable during the whole experiment.

The MRI acquisition protocol on the day of ketamine/saline injection included two rs-fMRI time series (pre-injection and post-injection at 30 min), FieldMap (before each rs-fMRI for correction of geometric distortions) and structural image (see Supplement for description) (Fig. 1). At 48 h post-injection the third rs-fMRI time series were acquired along with the FieldMap and structural image. The rs-fMRI T2*-weighted echo-planar imaging–free induction decay (EPI–FID) sequence was acquired using the following parameters: repetition time/echo time (TR/TE) 1500/17.5 ms, flip angle 60°, field of view 35 × 35 mm^2^, voxel dimension 0.365 mm, 30 coronal slices (ascending slice order), slice thickness 0.5 mm, 340 acquisitions over 8.5 min, and 8 dummy scans. A 3D double gradient echo FieldMap sequence was obtained using the following parameters: TR = 20 ms, short TE = 1.7 ms, and long TE = 5.7 ms. Structural brain image was acquired using a T2-weighted rapid acquisition with refocused echoes (RARE) sequence with the following parameters: RARE factor 16, TR/TE 1200/50 ms, flip angle 180°, the voxel dimension 0.15 mm, and acquisition time 23 min.

To compose the daily schedules of fMRI measurements, we used randomization according to the group (PC/NC), treatment (ketamine/saline), and time of the day. During the experiment, the investigator was blinded to the group assignment.

Image preprocessing was performed similarly to our previous studies^29,30^ and included the following steps: (1) correction of each EPI time series for magnetic field (B0) inhomogeneities and movement using “realign and unwarp” SPM function (SPM8: http://www.fil.ion.ucl.ac.uk/spm/software/spm8); (2) regressing out the estimated movement parameter vectors from each voxel (FSL, version 4.1. http://www.fmrib.ox.ac.uk/fsl); (3) filtering out respiratory and cardiac signals using Aztec software^31^; (4) slice-timing correction (SPM8); (5) spatial normalization to a rat brain template in Paxinos space^32^ (SPM8); (6) filtering out the cerebrospinal fluid signal from the normalized images (FSL); (7) frame-wise displacement (FD) and scrubbing to capture the remaining motion-related artifacts^33^, similarly to our previous study^30^, where we used an ellipsoid-like model instead of a spherical one, considering the non-spherical shape of rat brains compared to humans, and scrubbed all data frames with a FD > 0.03 mm and interpolated the missing frames using a linear interpolation; (8) the global signal regression; and (9) the band-pass filtering (0.01–0.1 Hz) (Analysis of Functional NeuroImages version 2)^34^. We note that due to a minimal amount of movement in rats, scrubbing had to be performed only in ~25% of datasets with removal of only 2–4 volumes in most of the animals. The effects of the preprocessing steps are presented in Supplementary Figs. S1 and S2.

### Graph theoretical analysis

#### Graph generation

Graphs consisted of Pearson correlation coefficients between time courses of 43 bilateral regions covering the whole brain^26^ and defined by Schwarz atlas^32^ (Fig. S3, Table S1). Edge weights of each subject and measurement were normalized, i.e. divided by their maximum correlation value within correlation matrices. The matrices were analyzed in Matlab using version 2015–01–25 of the freely available Brain Connectivity Toolbox^35^.

#### Integration of network metrics over a range of sparsity thresholds

Fully-connected weighted graphs were transformed into equi-sparse networks by retaining a fixed percentage of edges. To determine systematic effects on topological organization that would not depend on the choice of a single arbitrary threshold, we selected a range of thresholds. The lower threshold of 25% corresponded to the value at which networks for each subject and measurement were fully connected, meaning there were no infinite path lengths for any node, as was previously done^36^. At the upper threshold of 45% no negatively weighted edges were present, as previously described^26,29^. After thresholding the networks and calculating graph metrics for each of them, the area under the curve (AUC) was computed for each network metric. The groups were compared based on AUC parameters. The AUC method is sensitive to topological alterations in psychiatric disorders, and thus has been extensively used in brain network studies^2,37^.

#### Global and local metrics

For each network and sparsity level, we calculated five global metrics—characteristic path length, global clustering coefficient, small-world index, global efficiency, and local efficiency, and five local metrics—degree, strength, betweenness centrality, clustering coefficient, and local efficiency (see definitions in Supplement).

#### Correlation of behavior with network properties

We defined which regions differed in their baseline network properties between the NC and PC groups using permutation tests (10,000 permutations, *n* = 24 NC rats, *n* = 25 PC rats, *p* < 0.05) (Fig. 2) and then used Spearman tests to correlate those values that differed between the groups with individual behavioral parameters in the NC rats (*p* < 0.05).

#### Statistical analysis

To control for possible differences in overall connectivity strength, for each network a resampling algorithm created reference random networks with preserved degree and strength distributions (100 random networks)^38^. To normalize for individual baseline differences, we calculated differences of network properties, subtracting pre-injection values from post-injection (Fig. 1):

Δ1 = scan 2 − scan 1

Δ2 = scan 3 − scan 1

For each delta value, we calculated two-way ANOVA with “group” (NC/PC) and “treatment” (ketamine/saline), as factors (*p* < 0.05; false discovery rate correction *q* < 0.05 for the number of regions). We chose to analyse the second and the third time points separately as deltas (difference with the first time point) and in a rather qualitative fashion, because the differences to the first time point also include time effects due to prolonged anesthezia which would be difficult to interpret in a repeated measurements ANOVA with three factors.

## Results

### The NC rats express a shift toward network randomization and disrupted connectivity within cognitive, anxiogenic, and reward-related circuitries

In the baseline measurement (scan 1) NC rats, compared to PC rats, exhibited significantly lower global clustering coefficient (1.23 ± 0.01 versus 1.28 ± 0.01) and characteristic path length (1.05 ± 0.01 versus 1.09 ± 0.01), and higher global efficiency (0.96 ± 0.00 versus 0.93 ± 0.01) (Fig. 2), which represent a shift toward random organization.

The prefrontal and somato-motor regions in NC rats displayed lower degree, betweenness centrality and/or clustering, whereas the anxiogenic bed nucleus of stria terminalis and amygdala exhibited higher connectivity measures (Fig. 2, Table S2). Subcortical regions involved in working memory and cognitive flexibility, such as anterodorsal hippocampus, midline thalamus, and habenula, as well as reward-processing areas—ventral pallidum, nucleus accumbens, and ventral tegmental area—manifested increased strength in depressed rats (Fig. 2, Table S2).

### Escape deficit correlates with the connectivity metrics of cognitive, motor, anxiogenic, and reward-related nodes

Clustering coefficient of the anterodorsal hippocampus negatively correlated with *failure* (*r* = −0.43; *p* = 0.04) and *deficit pattern* (*r* = −0.43, *p* = 0.04). Somato-motor nodal properties inversely correlated with depressive behavior (Table S3). Amygdala, a key anxiogenic node, manifested a positive correlation of its strength with *failure pattern* (*r* = 0.49, *p* = 0.02) and *sum of latencies* (*r* = 0.45, *p* = 0.03). Reward-related ventral pallidum and ventral tegmental area exhibited positive correlation between their strength and efficiency, and *failure pattern* (*r* = 0.44, *p* = 0.03; *r* = 0.45, *p* = 0.03, respectively).

### Ketamine had a robust general effect on local metrics in both strains

**Fig. 3:**
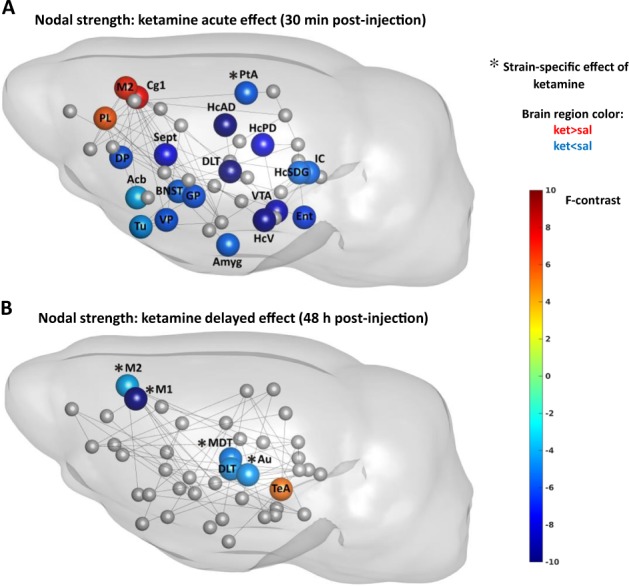
The acute (**a**) and delayed (**b**) effects of ketamine on nodal strength in the NC and PC groups. Here we show only the statistically significant effects of treatment (nodes without asterisk) and interaction of group and treatment (nodes with asterisk) on nodal strength from two-way ANOVA test. The effect of treatment (ketamine) had same direction in the NC and PC rats, whereas the effect of interaction of group and treatment had an opposite direction (for those cases we illustrate the direction in the NC group). Sphere color represents the *F*-statistic values from two-way ANOVA test for effects of treatment and interaction. The presence of connections (lines) between brain regions illustrate whether there was a change in inter-nodal correlation coefficients after ketamine in the NC rats versus saline condition. See legend of Fig. 2 for the abbreviations of brain regions

**Fig. 4:**
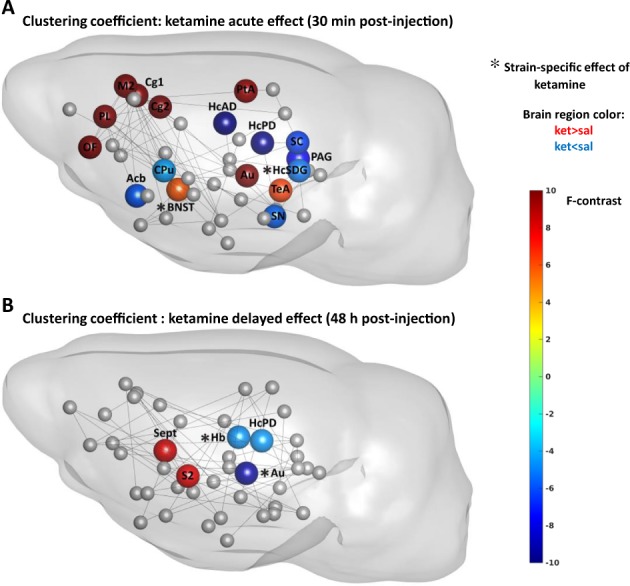
The acute (**a**) and delayed (**b**) effects of ketamine on nodal clustering coefficient in the NC and PC groups. See legend of Fig. 3 for the detailed description

Similarly to our previous study on healthy rats^29^, in both strains ketamine enhanced strength/clustering in the prefrontal regions, such as prelimbic (strength: treatment, *F*_1,45_ = 5.97, *p* = 0.02; clustering: treatment, *F*_1,45_ = 44.96, *p* < 0.001), cingulate (strength: treatment, *F*_1,45_ = 7.49, *p* = 0.01; clustering: treatment, *F*_1,45_ = 46.47, *p* < 0.001), orbitofrontal (clustering: treatment, *F*_1,45_ = 28.08, *p* < 0.001), and secondary motor cortices (strength: treatment, *F*_1,45_ = 6.91, *p* = 0.01) (Figs. 3 and 4; Table S4). An opposite change occurred in anterodorsal (strength: treatment, *F*_1,45_ = 10.08, *p* < 0.01; clustering: treatment, *F*_1,45_ = 22.58, *p* < 0.001) and posterodorsal hippocampus (strength: treatment, *F*_1,45_ = 8.00, *p* = 0.01; clustering: treatment, *F*_1,45_ = 15.35, *p* < 0.01) (Figs. 3 and 4; Table S4). Also, ketamine reduced strength in amygdala (treatment, *F*_1,45_ = 5.61, *p* = 0.02), and strength/clustering in nucleus accumbens (clustering: treatment, *F*_1,45_ = 5.96, *p* = 0.02), ventral pallidum (strength: treatment, *F*_1,45_ = 5.87, *p* = 0.02), and ventral tegmental area (strength: treatment, *F*_1,45_ = 8.61, *p* = 0.01) (Figs. 3 and 4).

### Ketamine had a short-lasting strain-unspecific effect on global metrics

**Fig. 5:**
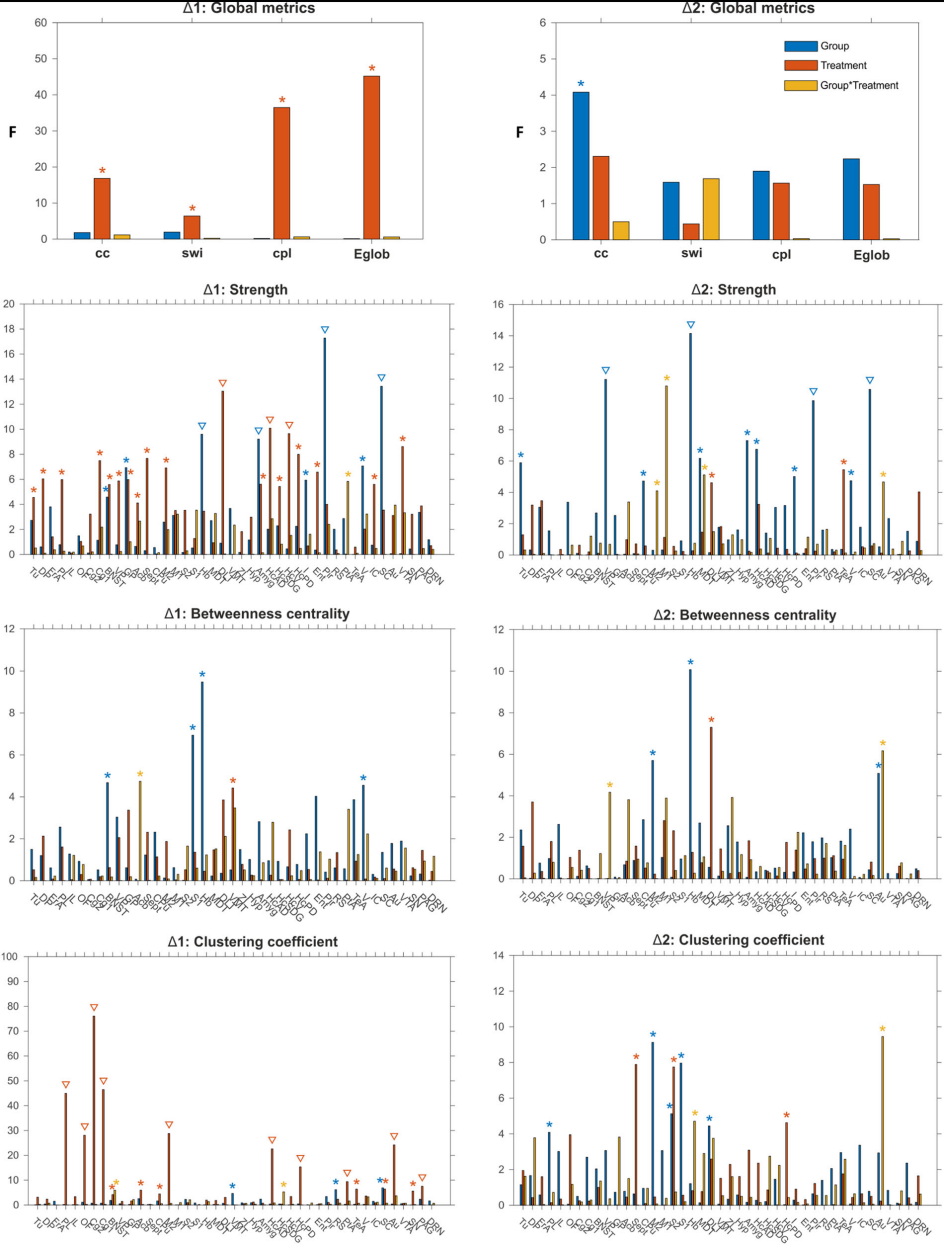
Effect of ketamine on global and local graph analytical properties at 30 min (Δ1) and 48 h (Δ2) post injection. The vertical bars are F-statistic values from two-way ANOVA test. Asterisk denotes significant results (*p* < 0.05), triangles (∇) signify results surviving false discovery rate correction (*q* < 0.05). cc clustering coefficient, cpl characteristic path length, Eglob global efficiency, swi small-world index

The change in global topological properties in response to ketamine was short-lasting (Fig. 5, Table S5), with no effects sustained 48 h later. The common response in both strains, as compared to respective control groups, was an increase in clustering coefficient (treatment, *F*_1,45_ = 16.84, *p* < 0.001) and path length (treatment, *F*_1,45_ = 36.48, *p* < 0.001), indicating increased network segregation and decreased integration, and a decrease in small-world index (treatment, *F*_1,45_ = 6.41, *p* = 0.01) and global efficiency (treatment, *F*_1,45_ = 45.20, *p* < 0.001).

### Ketamine had few strain-specific short-term effects

Depressed rats, contrary to PC rats, exhibited reduction of strength/clustering in nodes involved in cognitive processing, such as parietal association cortex (strength: interaction, *F*_1,45_ = 5.83, *p* = 0.02) and subiculum and dentate gyrus regions of the hippocampus (clustering: interaction, *F*_1,45_ = 5.28, *p* = 0.03) (Figs. 3 and 4). Also, ketamine raised clustering of bed nucleus of stria terminalis (interaction, *F*_1,45_ = 5.94, *p* = 0.02) in NC rats. The only node that showed the same direction of change in both groups, albeit in different extent, was the auditory cortex, exhibiting increased efficiency (interaction, *F*_1,45_ = 5.97, *p* = 0.02).

### Ketamine exhibited mainly strain-specific long-term effects

While most of ketamine’s acute effects were strain-independent, the delayed effects were mainly strain-specific (Figs. 3–5, Table S6) and expressed opposite directions between strains.

The strain-specific response included nodes that did not appear at acute stage, such as habenula displaying reduced clustering (interaction, *F*_1,45_ = 4.71, *p* = 0.04) and efficiency (interaction, *F*_1,45_ = 4.41, *p* = 0.04), and midline thalamus exhibiting reduced degree (interaction, *F*_1,45_ = 4.27, *p* = 0.04) and strength in depressed rats (interaction, *F*_1,45_ = 5.12, *p* = 0.03).

In addition, we observed diminished connectivity measures in the auditory (strength: interaction, *F*_1,45_ = 4.66, *p* = 0.04; betweenness centrality: interaction, *F*_1,45_ = 6.17, *p* = 0.02; clustering: interaction, *F*_1,45_ = 9.45, *p* < 0.01; efficiency: interaction, *F*_1,45_ = 11.23, *p* < 0.01) and primary motor cortices (degree: interaction, *F*_1,45_ = 11.42, *p* < 0.01; strength: interaction, *F*_1,45_ = 10.79, *p* < 0.01).

## Discussion

Regions that manifested the strongest strain baseline differences and showed correlation with depressive behavior, covered areas belonging to top 10% with the most abnormal connectivity features shared by four major depression types^3^. Ketamine effects on global topological organization were short-lasting and in opposite direction to patterns in depressed brain. Ketamine’s local action encompassed rapid strain-independent robust modification within the cognitive, sensory, subcortical emotion, and reward-related circuitry, including regions that exhibited correlation of their connectivity metrics with depressive behavior, and delayed strain-specific long-term normalization of connectivity measures for nodes mediating cognitive flexibility.

### Shift toward brain network randomization in depressed rats

Similarly to depressed patients, NC rats exhibited decreased global clustering^39–41^ and path length^2,40,42^, and enhanced global efficiency^2,40^, which signify decreased segregation and a shift toward brain network randomization. Two clinical studies report diminished global efficiency and increased characteristic path length^39,43^. However, the discrepancy of graph analytical findings in different studies is not surprising, given symptomatic heterogeneity of depression and the predominantly medicated state of patients, except two studies on drug-naïve subjects and patients after a medication wash-out^2,41^, both of which validate our results.

### Strain baseline differences in network local topology

NC rats manifested largely reduced prefrontal connectivity metrics, which is one of the most replicated findings in unmedicated depressed and PTSD patients^44–46^. This hypoconnectivity could result in blunted prefrontal top–down control over limbic system leading to enhanced amygdala activation and negative bias^47^. Consistently with this suggestion, we detected increased amygdala strength in the NC group, which positively correlated with depressive behavior. Furthermore, these changes might also contribute to enhanced freezing resistant to extinction in NC rats^17^, resembling persistent fear-related behavior in non-threatening situations in MDD and PTSD^48^, as consolidation of fear extinction occurs within the infralimbic-amygdala-midthalamic circuit^49^. Accordingly, NC rats exhibited increased strength for all regions of this circuit.

Another finding replicating human studies was increased connectivity measures of hippocampus and negative correlation of its metrics with depression severity^2,50^. Considered together with increased amygdala strength, it might reflect negative cognitive bias in which these regions facilitate recall of negative information^1^. On the other hand, finding lever to terminate footshock in escape test requires good navigation abilities and spatial memory, and since hippocampus mediates these functions^51^, our results might likewise mean that hippocampal hypoconnectivity could underlie impaired spatial cognition and memory across trials and consequently lead to worsened performance.

Next, habenula displayed higher degree, strength, centrality, and efficiency in the NC group, indirectly replicating habenula hyperactivity characteristic for depression^52,53^. Habenula mediates cognitive flexibility based on aversive events or context change^54^. We suggest that aberrant connectivity in habenula would result in impaired ability to switch behaviors adaptively despite negative outcomes. In addition, habenula suppresses motor activity^55^. Consistently, we found reduced somato-motor nodal properties in NC rats, as well as negative correlation of these regions connectivity with depressive behavior. Given that these regions control motor behavior, their diminished communication may directly affect behavioral performance.

Finally, NC rats demonstrated higher connectivity in reward-related regions, encompassing nucleus accumbens, ventral pallidum, and ventral tegmental area. Accumbens’ hyperconnectivity along with prefrontal hypoconnectivity might reflect two independent pathways triggering depression^56^. Accumbens’ primary output, ventral pallidum, processes both rewarding and aversive stimuli, and drives positive reinforcement^57^. Pallidal neurons exhibit elevated activity in depression^58^. In line with these data, we have detected positive correlation between pallidal strength and *failure pattern*. Furthermore, pallidal neurons enhance firing activity of habenula and ventral tegmental area^57,59^, both of which also displayed heightened strength and efficiency in NC rats.

### Ketamine’s acute strain-independent effects

Notably, the change in global topology in response to ketamine was short-lasting, with no effects sustained at 48 h. This leads to a conclusion that global topological reorganization is not required for ketamine’s long-term antidepressant effect, which rather builds upon fine-tuned topological modifications of specific nodes.

Ketamine acutely reduced strength of several regions that exhibited correlation of their metrics with depressive behavior, namely amygdala, anterodorsal hippocampus and ventral pallidum. Although the effect was strain-independent, it was in opposite direction to the baseline scan of NC rats. Modifying topological properties of these regions, ketamine presumably exerts anti-anxious, procognitive, and reinforcing effects. This normalization response did not sustain after 48 h, nevertheless, it might constitute a part of rapid antidepressant action.

Other common modifications included an increase in prefrontal strength and segregation, which we also detected earlier in healthy rats^29^ and is one of the most consistently replicated findings^56^, essential for ketamine treatment response and predicting treatment outcome^5,60^. It probably reflects ketamine-induced transient prefrontal glutamate surge and might underlie both dissociative and antidepressant effects^21^. Another effect that robustly replicates human studies was reduction of accumbens’ connectivity^56^. Reward-related nucleus accumbens and ventral tegmental area reduced their strength in response to ketamine, presumably reflecting ketamine’s positive reinforcement action.

Finally, motor cortex displayed increased strength, clustering, and efficiency after ketamine in both groups. This could explain a hyperlocomotion phenomenon after low-dose ketamine administration^61^.

Since all of these changes were in opposite direction to the baseline pattern in NC rats, we suggest that, although ketamine acted strain-independently, it was nevertheless capable to rapidly normalize topology within the cognitive, anxiogenic and reward-related regions in depressed rats, which could explain its quick antidepressant efficacy.

### Ketamine’s acute strain-specific effects

The parietal association cortex manifested strength change with opposite direction between strains. As this region integrates sensory information^62^, our result might reflect a difference in ketamine’s action between depressed and healthy individuals—interestingly, ketamine induces acute inner tension and somatic anxiety only in healthy individuals^63^.

Bed nucleus of stria terminalis and subiculum/dentate gyrus exhibited strain-specific contrary changes in clustering. Stria terminalis, as part of extended amygdala, is directly involved in expression of sustained anxiety^64,65^, therefore, this alteration may explain the feeling of heightened anxiety induced by ketamine specifically in healthy subjects^63^. Also, ketamine has differential effects on memory, transiently impairing it in healthy humans^66,67^, while improving in depressed patients^68^. As hippocampus participates in memory retrieval^69^, we suggest that strain-specific changes in hippocampal clustering could partially underlie this difference.

Finally, the auditory cortex displayed increased efficiency in both strains, differing only in extent and resembling response in healthy rats from our previous study^29^. Ketamine causes illusory auditory experiences^70^, which are generally associated with activation in the auditory cortex^71^. We reason that increased efficiency of the auditory cortex might reflect ketamine-induced auditory perceptual changes.

### Strain-specific long-term effects

Ketamine’s strain-specific long-term effects included normalization of connectivity measures for habenula and midline thalamus. Importantly, these two regions along with hippocampus, whose connectivity was also normalized at both time points, mediate cognitive flexibility^72,73^—a skill profoundly impaired in MDD and PTSD^73,74^, when individuals are unable to process contextual information, distinguish contextual cues in safe versus threatening situations and to modulate fear and emotional response in non-threatening environment. Cognitive flexibility critically depends on contextual encoding and memory processing^54^, which is also impaired in MDD and congenital learned helplessness model^75^. CA1–CA3 regions of the hippocampus, covered by the anterodorsal and posterodorsal seeds in our study, create distinct representation pattern for each situation^69^, thus allowing avoidance of context overgeneralization characteristic for depressed state. Midline thalamus is a partner of hippocampus in forming memories^51,72^. Ketamine improves memory in depressed patients, in contrast to healthy subjects;^68^ also, it ameliorates cognitive flexibility deficit in chronically stressed rats at the same dose like in our study^76^. Since we utilized cognitive model of depression, we suggest that ketamine mediates its procognitive effects by normalizing the disrupted wiring within the habenula-midthalamic-hippocampal cognitive circuitry, which might be a key imaging correlate of its long-term effect.Also, our result of reduced connectivity metrics of habenula may correspond to ketamine-induced reduction of habenula metabolism in patients with treatment-resistant depression^77^ and inhibition of habenula burst firing activity in learned helplessness model^78^, both producing antidepressant action.

Ketamine had a strain-specific effect on the auditory cortex, consistently reducing almost all of its connectivity metrics in NC rats and retaining them raised in PC rats. Since in the baseline scan depressed rats did not show differences in the auditory cortex connectivity measures, this action does not appear to constitute a part of ketamine’s antidepressant effect, but might rather represent a lasting modification of the auditory system function. Most studies report a ketamine-induced acute reduction of auditory event-related potentials parameters and mismatch negativity amplitude^79,80^, which might underlie changes in auditory perception, however, the duration of this effect remains to be investigated. On the other hand, since anxiety is characterized by hypervigilance and scanning for potentially threatening auditory cues, downregulation of auditory system might be part of calming effect.

### Limitations

We did not test antidepressant effects of ketamine, since it would greatly interfere with the whole experimental setup, when done between fMRI sessions, and at later stage two scanning sessions would affect test results as stress factors. Previous works already revealed ketamine antidepressant action in learned helplessness behavior^7,81,82^. Although it remains to be demonstrated that our findings would predict treatment response, they illustrate the potential of these empirically defined circuits to serve as benchmarks of ketamine’s response.

## Conclusion

Using the negative cognition model of depression, we identified ketamine’s qualitatively distinct action over time. The rapid topological modification within the cognitive, sensory, and subcortical emotion and reward-related circuitry could explain both dissociative and antidepressant effects. The delayed strain-specific normalization of disrupted connectivity within the habenula-midthalamic-hippocampal circuitry presumably reflects ketamine’s procognitive efficacy observed only in depressed patients. This finding is especially valid, as we used model representing cognitive aspect of depression and might serve as translational imaging correlates of antidepressant response in preclinical testing.

## Supplementary information

Supplementary.
