# Supplementary material for: Differences between ketamine’s short-term and long-term effects on brain circuitry in depression
Source: Transl Psychiatry. 2019 Jun 28;9:172. doi: 10.1038/s41398-019-0506-6 (PMC6599014; doi:10.1038/s41398-019-0506-6)
Supplement: Supplementary file 1 — Supplementary. [file 41398_2019_506_MOESM1_ESM.doc]

**Supplemental Material for:**

N. Gass, R. Becker, J. Reinwald, A. Cosa-Linan, M. Sack, W. Weber-Fahr, B. Vollmayr, A. Sartorius.

**Differences between ketamine’s short-term and long-term effects on brain circuitry in depression**

**Experimental procedures**

**Test for escape behavior.** Since in later (more than 20) generations an escape deficit was found even in absence of prior inescapable shocks, the behavioral procedure was performed without uncontrollable shocks to minimize animal suffering 1. The chambers had inside dimensions of 48.5x30x21.5 cm with the floor constructed of steel rods (6 mm in diameter, 20 mm apart). A 35x35 mm lever was positioned on one side of the boxes. The experiment was controlled by an IBM compatible 4/86 computer (boxes, shock generator and controlling program obtained from TSE, Bad Homburg, Germany). The test consisted of 10 trials of 0.8 mA current, each lasting 60 s (inter-trial time 24 s), if the animal did not stop the current by pressing the lever. The latency to stop a trial by pressing the lever was recorded. For analysis we chose sum of latencies from trials 3-10, since during the first two trials rats respond unspecifically with hyperlocomotive agitation 1.

**MRI acquisition.** The rs-fMRI experiments were carried out at a 9.4 Tesla MRI scanner (Bruker BioSpec, Ettlingen, Germany) with Avance III hardware, BGA12S gradient system (maximum strength 705 mT/m) and Paravision 6 software, using the linear whole-body volume transmitter coil combined with an anatomically shaped four-channel receive-only coil array for transmission and reception.

**Graph theoretical analysis.** For each network and sparsity level we calculated five global metrics: (1) global clustering coefficient = the average of all nodal clustering coefficients, (2) characteristic path length = the average shortest path length, (3) small-worldness index = the ratio of global clustering coefficient and characteristic path length, (4) global efficiency = the average inverse shortest path length, (5) local efficiency = the average of local efficiency values for all nodes. For the nodal analysis we computed five local graph metrics: (1) degree = the number of edges connecting a given node to other nodes, (2) strength = the sum of nodal edges weights, (3) betweenness centrality = the percentage of all shortest paths in the network containing the given node, (4) clustering coefficient = the number of edges between the neighbors of a given node normalized to the maximum number of possible edges, (5) local efficiency = global efficiency computed on a node’s neighborhood.

**Structural image**

**Before B0 correction**

**After B0 correction**


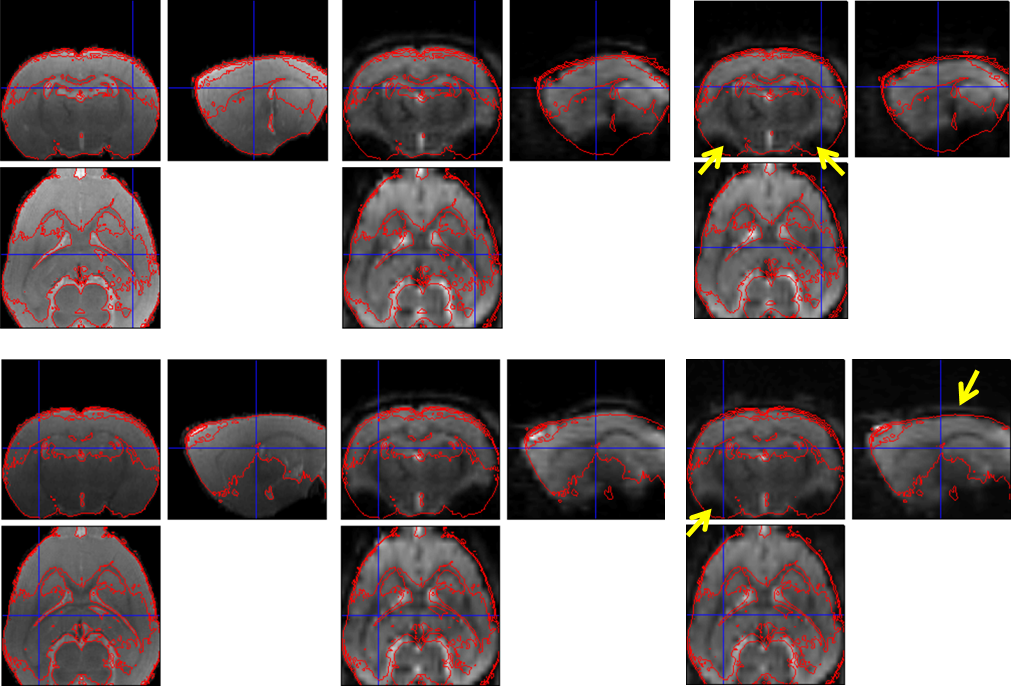


**Figure S1.** The effects of the B0 correction on EPI image. The purpose of this correction is to reduce the EPI-inherent geometrical distortions due to inhomogeneity of the magnetic field, thus improving the spatial accuracy of the data. Yellow arrows indicate those parts of the image, where the B0 correction resulted in filling up the missing parts of the brain (top panel) or getting rid of the artificial bump over the brain surface (low panel).


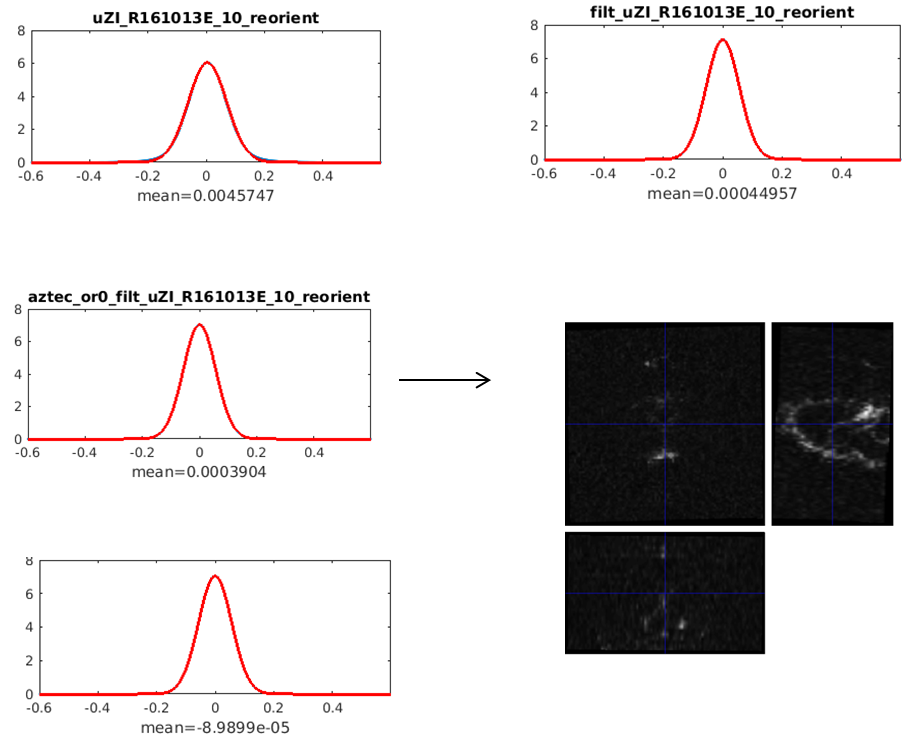


1. **B0 correction**

**(C) Physiological signal regression**

**(B) Motion regression**

**(D) The explained variance of the complete physiological signal regression**

**(E) Global signal regression**

**Figure S2.** The effects of pre-processing steps on the mean voxel correlation value. Systematic noise due to movement or physiological signal shifts a mean voxel correlation value to the right due to spurious hyper-correlations, whereas filtering out the noise sources in pre-processing routine shift the signal to the left. A mean value under each plot shows the mean voxel correlation value. Motion regression (B) shifted the value to the left, as compared to (A), since motion can introduce coherent BOLD changes across the whole brain. Plot (D) demonstrates the effect of the respiratory and cardiac signal regression using Aztec – the bright spots along the border of the brain and ventricles illustrate the explained variance of the complete physiological signal regression. Global signal regression (E) shifts the signal further to the left.

**
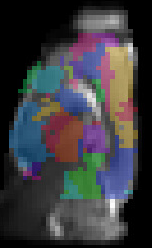
**

**Figure S3.** Regions of interest used for analysis overlaid on the mean EPI image.

**Table S1.** An overview of the regions of interest, their volume and percentage of their coverage the mean EPI image.

| **Brain regions** | **Number of voxels** | **Percentage of voxels within the brain** |
| --- | --- | --- |
| **Tu** | 313 | 94 |
| **DP** | 35 | 100 |
| **FrA** | 238 | 94 |
| **PL** | 225 | 100 |
| **IL** | 53 | 100 |
| **OF** | 553 | 93 |
| **Cg1** | 243 | 100 |
| **Cg2** | 134 | 100 |
| **BNST** | 125 | 100 |
| **VP** | 110 | 100 |
| **GP** | 192 | 100 |
| **Acb** | 336 | 100 |
| **Sept** | 316 | 100 |
| **M1** | 1171 | 100 |
| **M2** | 692 | 100 |
| **S1** | 2969 | 100 |
| **S2** | 528 | 100 |
| **Hb** | 36 | 100 |
| **MDT** | 383 | 100 |
| **DLT** | 1080 | 100 |
| **VMT** | 83 | 100 |
| **ZI** | 120 | 100 |
| **Hyp** | 922 | 100 |
| **Amyg** | 577 | 57 |
| **HcAD** | 422 | 100 |
| **HcSDG** | 454 | 100 |
| **HcV** | 518 | 72 |
| **HcPD** | 760 | 100 |
| **I** | 741 | 94 |
| **Ent** | 45 | 52 |
| **Pir** | 1392 | 70 |
| **RS** | 678 | 100 |
| **PtA** | 253 | 100 |
| **TeA** | 249 | 100 |
| **V** | 1096 | 100 |
| **IC** | 61 | 100 |
| **SC** | 465 | 100 |
| **Au** | 582 | 100 |
| **VTA** | 85 | 100 |
| **SN** | 143 | 100 |
| **PAG** | 162 | 100 |
| **DRN** | 8 | 100 |

**Results**

**Behavioral results.** Compared to the PC rats, the NC rats exhibited a higher *sum of latencies* (375.36±23.05 vs 243.24±21.50; p<0.001) (Fig. 2), similarly to the previous reports 1, 2. They also demonstrated a higher value for *failure pattern* (5.88±0.60 vs 2.72±0.49; p<0.001) and for *deficit pattern* (7.63±0.53 vs 5.68±0.45; p<0.01) (Fig. 2).

**Table S2.** Regions showing altered (increased ↑ or decreased ↓) local metrics in rats bred for negative (NC) and positive (PC) cognitive state, as compared to the control (saline) group. See legend of the Figure 2 for the abbreviations of brain regions.

| **Brain regions** | **Permutation p-value** | | | | |
| --- | --- | --- | --- | --- | --- |
|  | **Degree** | **Strength** | **Betweenness**  **centrality** | **Clustering**  **coefficient** | **Local efficiency** |
| **Tu** |  | ↑ 0.01 |  |  |  |
| **DP** |  |  |  | ↓ 0.01 | ↓ 0.04 |
| **FrA** |  | ↑ <0.01 |  |  |  |
| **PL** | ↓ 0.03 |  | ↓ 0.02 | ↓ <0.001 | ↓ <0.001 |
| **IL** | ↓ <0.001 |  | ↓ 0.03 |  |  |
| **OF** |  |  |  | ↓ 0.02 |  |
| **Cg1** |  |  | ↓ 0.03 | ↓ 0.02 | ↓ 0.04 |
| **Cg2** | ↓ 0.01 |  |  | ↓ 0.01 | ↓ 0.04 |
| **BNST** |  | ↑ <0.001 | ↑ 0.02 | ↑ 0.04 | ↑ 0.01 |
| **VP** | ↑ 0.01 | ↑ <0.001 |  |  |  |
| **GP** | ↑ <0.01 | ↑ <0.001 |  | ↑ 0.03 | ↑ <0.01 |
| **Acb** |  | ↑ 0.01 |  |  |  |
| **M1** |  |  | ↓ <0.001 | ↓ <0.001 | ↓ <0.001 |
| **M2** |  |  |  | ↓ <0.001 | ↓ 0.01 |
| **S1** |  |  |  | ↓ <0.001 | ↓ <0.01 |
| **S2** |  |  |  | ↓ 0.01 | ↓ <0.01 |
| **Hb** | ↑ 0.03 | ↑ <0.001 | ↑ <0.001 |  | ↑ 0.04 |
| **MDT** |  | ↑ <0.001 |  |  |  |
| **DLT** |  |  |  | ↓ 0.02 | ↓ 0.01 |
| **VMT** |  | ↑ <0.01 |  |  | ↑ 0.02 |
| **ZI** |  | ↑ 0.01 |  |  |  |
| **Hyp** |  | ↑ 0.01 |  |  |  |
| **Amyg** |  | ↑ <0.01 |  |  |  |
| **HcAD** |  | ↑ <0.001 |  | ↑ 0.03 | ↑ 0.02 |
| **HcV** |  | ↑ 0.01 |  |  |  |
| **HcPD** |  | ↑ 0.03 |  |  |  |
| **I** | ↑ 0.03 | ↑ <0.001 |  |  |  |
| **Ent** |  | ↑ 0.01 |  |  |  |
| **Pir** |  | ↑ <0.001 |  |  | ↑ 0.04 |
| **RS** | ↓ 0.03 |  | ↓ 0.02 |  |  |
| **PtA** |  | ↑ 0.01 |  |  |  |
| **TeA** | ↓ 0.03 |  | ↓ 0.02 |  | ↓ 0.02 |
| **V** | ↓ 0.02 |  |  |  |  |
| **IC** | ↑ <0.01 | ↑ <0.001 |  |  |  |
| **SC** |  | ↑ <0.001 | ↑ 0.03 | ↑ 0.03 | ↑ 0.02 |
| **VTA** |  | ↑ <0.001 |  | ↑ 0.02 | ↑ 0.01 |
| **SN** |  | ↑ 0.01 |  |  |  |
| **PAG** |  | ↑ <0.01 |  | ↑ 0.02 |  |
| **DRN** |  | ↑ 0.01 |  |  |  |

**Table S3.** Regions showing correlation between their local topological properties and the behavioral parameters in rats bred for negative cognitive state. See legend of the Figure 2 for the abbreviations of brain regions.

| **Brain regions** | **Correlation coefficient *r*; *p-value*** | | |
| --- | --- | --- | --- |
|  | **Failure pattern** | **Deficit pattern** | **Sum of latencies** |
| *degree* | | | |
| **I** |  | -0.43; 0.04 |  |
| **SC** |  | -0.45; 0.03 |  |
| *strength* | | | |
| **VP** | 0.44; 0.03 |  |  |
| **Amyg** | 0.49; 0.02 |  | 0.45; 0.03 |
| *clustering coefficient* | | | |
| **M1** | -0.47; 0.02 | -0.48; 0.02 | -0.58; <0.01 |
| **S1** | -0.53; 0.01 | -0.47; 0.02 | -0.52; 0.01 |
| **HcAD** | -0.43; 0.04 | -0.43; 0.04 |  |
| **SC** |  |  | -0.42; 0.04 |
| *local efficiency* | | | |
| **M1** | -0.42; 0.04 | -0.44; 0.03 | -0.53; 0.01 |
| **SC** |  | -0.47; 0.02 | -0.44; 0.03 |
| **VTA** | 0.45; 0.03 |  |  |

**Table S4.** Acute (30 min post-injection) effects of ketamine on local metrics (increase ↑ or decrease ↓) in rats bred for negative (NC) and positive (PC) cognitive state, as compared to the control (saline) group. Triangle (^∇^) signifies values which survived FDR correction (q<0.05). See legend of the Figure 2 for the abbreviations of brain regions.

| **Brain regions** | **p-value** | | | | |
| --- | --- | --- | --- | --- | --- |
|  | **Degree** | **Strength** | **Betweenness**  **centrality** | **Clustering**  **coefficient** | **Local efficiency** |
| **NC-ketamine vs NC-saline** | | | | | |
| **PL** |  |  |  | ↑ <0.001^∇^ | ↑ <0.001^∇^ |
| **IL** | ↑ 0.01^∇^ |  |  |  |  |
| **OF** |  |  |  | ↑ <0.01^∇^ | ↑ <0.001^∇^ |
| **Cg1** |  |  |  | ↑ <0.001^∇^ | ↑ <0.001^∇^ |
| **Cg2** |  |  |  | ↑ <0.001^∇^ | ↑ <0.001^∇^ |
| **Acb** |  | ↓ 0.01 |  |  | ↓ 0.02 |
| **M1** |  | ↓ 0.01^∇^ |  |  |  |
| **M2** |  |  |  | ↑ <0.01^∇^ | ↑ 0.01^∇^ |
| **S1** |  | ↓ 0.04 |  |  |  |
| **DLT** |  | ↓ 0.02^∇^ |  |  |  |
| **VMT** |  |  | ↑ 0.01^∇^ |  |  |
| **HcAD** |  | ↓ <0.01^∇^ |  | ↓ <0.01^∇^ | ↓ <0.001^∇^ |
| **HcSDG** |  | ↓ 0.03 |  |  |  |
| **HcV** |  | ↓ <0.01^∇^ |  |  |  |
| **HcPD** |  | ↓ 0.02 |  | ↓ 0.01^∇^ | ↓ <0.001^∇^ |
| **TeA** | ↑ 0.03 |  |  |  |  |
| **V** |  | ↓ 0.03 |  |  |  |
| **IC** |  | ↓ 0.04 |  |  |  |
| **SC** |  |  |  | ↓ 0.02^∇^ | ↓ 0.02 |
| **Au** |  |  |  | ↑ 0.04 |  |
| **VTA** |  | ↓ <0.01^∇^ |  |  |  |
| **PC-ketamine vs PC-saline** | | | | | |
| **Tu** |  |  |  |  | ↓ 0.03 |
| **PL** |  | ↑ 0.04 |  | ↑ <0.001^∇^ | ↑ <0.001^∇^ |
| **OF** |  |  |  | ↑ <0.001^∇^ | ↑ <0.001^∇^ |
| **Cg1** |  | ↑ <0.01^∇^ |  | ↑ <0.001^∇^ | ↑ <0.001^∇^ |
| **Cg2** |  |  |  | ↑ <0.001^∇^ | ↑ <0.001^∇^ |
| **BNST** |  |  |  | ↓ <0.01^∇^ | ↓ <0.01^∇^ |
| **GP** |  | ↓ 0.02^∇^ |  |  | ↓ 0.01 |
| **M2** |  | ↑ 0.01^∇^ |  | ↑ <0.001^∇^ | ↑ <0.001^∇^ |
| **DLT** |  | ↓ 0.01^∇^ | ↓ 0.02 |  | ↓ 0.01^∇^ |
| **HcAD** |  |  |  | ↓ 0.04 | ↓ <0.01^∇^ |
| **HcSDG** |  |  |  | ↑ 0.04 |  |
| **HcPD** |  |  |  | ↓ 0.01^∇^ | ↓ <0.01^∇^ |
| **Ent** |  | ↓ 0.04 |  |  | ↓ 0.02 |
| **Pir** |  | ↓ 0.01^∇^ |  |  |  |
| **PtA** |  |  |  | ↑ <0.01^∇^ | ↑ 0.01^∇^ |
| **TeA** |  |  |  | ↑ 0.02 |  |
| **V** |  |  |  | ↑ 0.01 |  |
| **Au** |  | ↑ 0.01 |  | ↑ <0.001^∇^ | ↑ <0.001^∇^ |
| **SN** |  |  |  |  | ↓ 0.02 |

**Table S5.** Acute (30 min post-injection) effects of ketamine on global metrics (increase ↑ or decrease ↓) in rats bred for negative (NC) and positive (PC) cognitive state, as compared to the control (saline) group. Triangle (^∇^) signifies values which survived FDR correction (q<0.05).

| **p-value** | | | | |
| --- | --- | --- | --- | --- |
| **Clustering coefficient** | **Small-world index** | **Path length** | **Global efficiency** | **Local efficiency** |
| **NC-ketamine vs NC-saline** | | | | |
| ↑ 0.04 | ↓ 0.04 | ↑ <0.001^∇^ | ↓ <0.001^∇^ | >0.05 |
| **PC-ketamine vs PC-saline** | | | | |
| ↑ <0.001^∇^ | >0.05 | ↑ <0.001^∇^ | ↓ <0.001^∇^ | >0.05 |

**Table S6.** Delayed (48 h post-injection) effects of ketamine on local metrics (increase ↑ or decrease ↓) in rats bred for negative (NC) and positive (PC) cognitive state, as compared to the control (saline) group. Triangle (^∇^) signifies values which survived FDR correction (q<0.05). See legend of the Figure 2 for the abbreviations of brain regions.

| **Brain regions** | **p-value** | | | | |
| --- | --- | --- | --- | --- | --- |
|  | **Degree** | **Strength** | **Betweenness**  **centrality** | **Clustering**  **coefficient** | **Local efficiency** |
| **NC-ketamine vs NC-saline** | | | | | |
| **DLT** |  | ↓ 0.02 |  | ↓ 0.02^∇^ |  |
| **TeA** | ↑ <0.01^∇^ |  |  |  |  |
| **Au** |  |  |  |  | ↓ 0.02^∇^ |
| **DRN** | ↑ 0.01 |  |  |  |  |
| **PC-ketamine vs PC-saline** | | | | | |
| **DP** |  |  |  |  | ↓ 0.03 |
| **OF** |  |  |  | ↑ 0.03 |  |
| **M1** | ↑ 0.01^∇^ | ↑ <0.01^∇^ | ↑ 0.01 |  |  |
| **S2** |  |  |  | ↑ 0.01^∇^ | ↑ 0.03 |
| **Hb** |  |  |  | ↑ 0.03 |  |
| **MDT** |  | ↑ 0.02^∇^ |  |  |  |
| **TeA** |  |  |  | ↑ 0.04 | ↑ 0.04 |
| **Au** |  |  |  | ↑ 0.01^∇^ | ↑ 0.02^∇^ |

**References**

1. Richter SH, Sartorius A, Gass P, Vollmayr B. A matter of timing: Harm reduction in learned helplessness. Behav Brain Funct 2014; 10: 41,9081-10-41.

2. Gass N, Becker R, Schwarz AJ, Weber-Fahr W, Clemm von Hohenberg C, Vollmayr B, et al. Brain network reorganization differs in response to stress in rats genetically predisposed to depression and stress-resilient rats. Transl Psychiatry 2016; 6: e970.
